# Strengthening the traditional medicine regulation, education and practice in Nigeria

**DOI:** 10.4314/ahs.v24i3.50

**Published:** 2024-09

**Authors:** Sanjoy Kumar Pal, Lawal Isa

**Affiliations:** 1 Skyline University Nigeria, Department of Biological Sciences; 2 Skyline University Nigeria, School of Basic Medical Sciences

## Abstract

**Background:**

Despite tremendous advances in medical sciences still a large section of the population in rural areas is heavily dependent on indigenous and Traditional Medicine (TM) for treatment. Moreover, there has been a resurgence of interest in the use of Complementary and Alternative Medicine (CAM), globally.

**Objective:**

To review the overall regulator structure for the control of TM and the course curriculum for TM education in Nigeria.

**Method:**

Information on CAM and TM was collected via online search.

**Results:**

Responding to this unprecedented situation government of many countries have taken proactive steps to streamline and regulate the practitioners of CAM and TM in their countries as mandated by WHO. In many African countries because of the availability of research and training programs many traditional herbal medicines are now being sold over the counter. However, the situation is different in Nigeria, even though TM is very popular across the country, structured institutionalized teaching and research activity on TM are inadequate.

**Conclusion:**

Health is a key component in determining the Human Development Index (HDI) of a country. CAM and TM practitioner may play an important role in improving the HDI score of the country. Government-sponsored education & training program for TM practitioners is crucial.

## Introduction

Globally, there has been a resurgence of interest in the use of Complementary and Alternative Medicine (CAM) and it is the most popular form of therapy in African households^1^. A large proportion of the African population uses CAM as a substitute for conventional medicine^2^. CAM has received renewed attention and interest around the world, particularly among patients suffering from chronic diseases^3^. The clinical outcome of management of chronic illness entirely depends on how religiously one follows the prescribed conventional treatment; however, very negligible importance is given to knowing about the concomitant use of alternative medicines^4^. It has been observed that the patients are not very comfortable to disclose about CAM usage and in some situations, the treating clinicians have limited knowledge about CAM use; hence, they are apprehensive to initiate discussion on CAM 5. This is because conventional doctors are not educated about various CAM practices during their medical studies^6^. t is very crucial for conventional clinicians to understand the factors influencing the use of TM/CAM and their interaction with orthodox medicine^3^. There are many complementary therapies such as yoga, exercises, meditation, etc., which are helpful in the management of various lifestyle-oriented diseases like obesity, hypertension, heart problem, diabetes, etc.

‘Indigenous Medicine Practice’ in Nigeria is a traditional heritage, and it is practiced in all parts of the country^7^ and enjoys considerable support from the populace^8^. Various forms of therapies that are popular include herbals, homeopathy, massage, mud bath, spinal manipulation, mind & spirit therapy, hydrotherapy, wax bath, diet, bone setting, psychotherapy, etc^9^. Even though a substantial proportion of the population in Nigeria heavily depends on alternative and traditional medicine for health care; however, institutionalized CAM education is conspicuously missing in this country 4,10. Very few Nigerian universities are having full-fledged courses in CAM or Traditional Medicine. Few universities that have some herbal medicine courses are mostly confined to the pharmacology department. Contrary to this, countries like India, China, and the USA have well-structured courses on alternative medicine which are officially recognized, institutionally taught, and professionally practiced. Many alternative medicine practices have blossomed into orderly-regulated systems of medicine that include: Ayurveda, Traditional Chinese Medicine, Homeopathy, Chiropractic, Unani, Siddha, Naturopathy, Yoga, etc.^11^

## Regulation of CAM

The regulation of CAM practice is really a challenge because of the nature of its diversity. In different European countries various CAM treatments are regulated differently^12^. While in most countries the law suggests that the delivery of health care should be controlled by biomedically-trained professionals; however, alternative healers operate very openly, and many don't even have any proper training 13. The impending harm that a fraudulent CAM practitioner may cause is a reckless decision, wrong diagnosis, misleading information, improper treatment, etc^14^. The consumption of CAM and traditional medicine in rural areas is a significant contemporary health care issue 15. Due to the availability of proper research facility and education in many African countries viz. South Africa, Sierra Leone, and Tanzania traditional medicine products are now being sold over the counters. In Ghana, many TM products are now included in the list of National Essential Medicine 16, this effort was achieved through proper regulatory processes.

According to an estimate, the global market of CAM products is expected to reach US $5 trillion by 2050^17^. As the use of CAM has skyrocketed, many governments across the world have now recognized the need for comprehensive regulation of CAM practices. The World Health Organization (WHO) has also called for the increased statutory regulation of traditional and complementary medicine practitioners and practices^18^. Responding to this unprecedented situation the Ministry of Health in Saudi Arabia has recently established National Center for Complementary and Alternative Medicine (NCCAM). This center is responsible to look after all activities related to complementary medicine. One of the most important roles of NCCAM is setting the rules and criteria for regulating, monitoring, and supervising CAM. Currently, acupuncture, cupping therapy, chiropractic, osteopathy, and naturopathy are regulated through the NCCAM 19. The Government of India regulates various CAM practices through its ministry of AYUSH. The name AYUSH is derived from the names of the alternative healthcare systems covered by the ministry: Ayurveda, Yoga & Naturopathy, Unani Siddha, and Homeopathy. AYUSH is solely responsible for regulation, research, and propagation of traditional medicine systems in the country 20. In the USA the regulation activities of various CAM practices are done by the Governmental agency, the National Center for Complementary and Integrative Health (NCCIH), which was established in 1991^21^. In China, the traditional herbal preparations are regulated by the National Medical Products Administration (NMPA). Strict rules are followed in the manufacturing process of herbal products which is at par with the conventional formulations. For the practice of acupuncture and Chinese medicine one have to obtain a certificate either (i) by studying at a regular medical school; (ii) with a teacher who has enough experience or professional qualification; (iii) through special training programs for countryside doctor 22. Obviously, there is growing global interest in the use of traditional medicine and to ensure safety, control abuse and standardize practice it is necessary for countries to develop national policies for proper regulation. Predisposing circumstances, enabling factors, need factors, and their different components are the three main elements that influence whether CAM is chosen as the medical system in a given circumstance^23^. However, it is crucial to check whether decisions regarding health care are being made in accordance with available evidence when using a pluralistic model of health care delivery. The ultimate goal of any pluralistic health care policy should be to offer patients a competitive yet effective range of medical options that are tailored to their own requirements and cultural beliefs. The final result of a disease may be significantly impacted by factors influencing the healthcare decisions. Under the impact of driving circumstances, a poor decision could lead to subpar treatment, delayed responses, higher therapy costs, and harms that may be irreparable.

## Regulation of CAM in Nigeria

In Nigeria at the moment, it is difficult to assert that there is a specific regulatory body or agency with primary responsibility for the overall control of the traditional methods of health care delivery. Though, recently the Federal Government has created a Department of Traditional Medicine in the Ministry of Health to initiate and conduct research into the discovery and application of traditional medicines^24^. There is no distinct legal framework established by the Federal Government to monitor or control the practice of traditional medicine. Moreover, laws on traditional methods of health delivery are insufficient, and the implementation of related legislation or regulation is weak 7. A study focused on policy and regulation of traditional medicine in Nigeria found that a majority of herbal medicines that were sold in Nigeria were not done by a licensed practitioner^25^. Nigeria need to take strong lift from the fact that despite many challenges, countries of the European Union (EU), India, China, United Kingdom, Japan, South Korea, Malaysia, and Australia have made giant strides to ensure the safe use of herbal medicines. A similar type of strong regulation is also required in Nigeria. This can only be attained when there are various offices/departments are created at the National and state level across the country to oversee the activities and regulation of traditional, CAM, and herbal medicines. Attempts are now being made by the Federal Government to establish traditional medicine departments in all 36 states of Nigeria 26. A Council for Traditional, Alternative, and Complementary Medicine Practice in Nigeria has also been recently approved 27.

**Figure 1:**
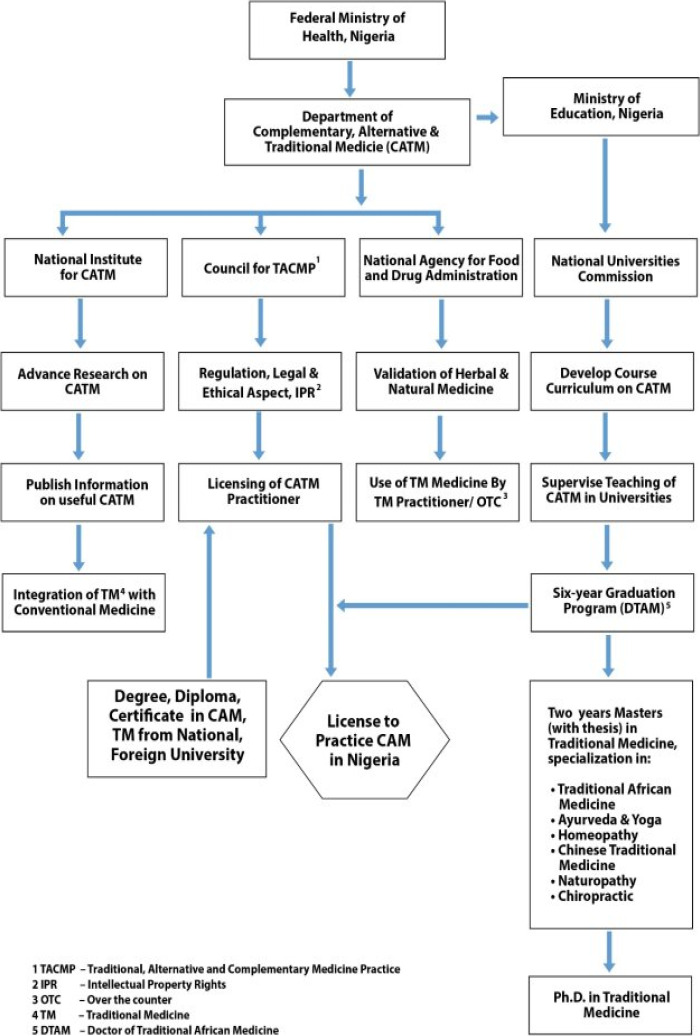
Proposed organizational flowchart related to regulation of traditional medicine in Nigeria

To promote the use of evidence-based alternative medicine in the country the full organizational structure along with the vision and mission related to traditional medicine must be made clear to the common public. A governmental portal should be created for dissemination of CAM rules and regulation in Nigeria. In this review an attempt has been made to outline the main structural organization that may play an important role in the regulation, and promotion of traditional and CAM medicine in Nigeria (Figure 1).

License to Sale Product of Herbal Medicine in Nigeria Herbal medicine is an integral part of traditional medicine 28. However, the widespread use of herbal medicine globally does not guarantee the safety of the product^29^. Good quality medicine can be considered as an important hallmark in health care delivery. However, as many herbal preparation are still not standardizd, availability sometimes become an issue 30. Moreover, rigorous clinical trials are virtually not available for many chronic illnesses treated exclusively with alternative medicines so the effectiveness of many CAM therapies are inefficiently determined. As there are fewer clinical studies conducted with herbal medicine visa-a-vis conventional medicine, hence, safety and effectiveness of traditional herbal products remain a big concern. Many herbal medicine manufacturing companies still do not strictly adhere to the stringent guidelines prescribed by the WHO. Many ethnomedicinal preparations that have a long history of use are now undergoing clinical testing worldwide. However, these experimental efforts will still require clinical safety standardization which are to be guided by local (country) and international regulatory conditions. In Nigeria, the National Agency for Food, Drugs, Administration, and Control (NAFDAC) is in charge providing such regulatory conditions and it is now focusing its effort in testing many ethnomedicinal preparations that are currently in use in the market^25^. NAFDAC plays a vital role in regulating the activities of traditional/alternative medicine practitioners, mainly in the aspect of herbal medicines 31. NAFDAC plays the same role as the Food and Drug Administration (FDA) in the USA or National Medical Products Administration (NMPA) in China. Under these agencies full registration of herbal product is only granted after the product had shown satisfactory efficacy and convincing safety profile. To get approval for commercial use all herbal products must undergo the same rigorous clinical trial as that of conventional medicine so that the harm to the patients is minimized. The coverage of a large number of herbal products in Nigerian streets by NAFDAC is far from being adequate as many herbal medicine practitioners do not present their products for test and efforts being made to reduce the circulation of such products is completely unclear.

## License to Practice Traditional Medicine & CAM

The licensing of traditional medicine practitioners should receive an equal and necessary attention as the regulation of the products. Hence, it should be made mandatory that whoever wishes to practice alternative medicine should first possess a valid license provided by a recognized government agency in any given country (such as Nigeria). Presently, there is no any national agency saddled with the responsibility of licensing practitioners of traditional medicine.

This paper is proposing for establishment of an agency to offer such license or an expanded mandate should be given to the Pharmaceutical council. To qualify for a license individuals can be graded according to the quality of training received, experience and other criteria to be determined by the licensing body. Typically, individuals may be considered if they: (i) study at a recognized university/polytechnic or any recognized tertiary institution (ii) pass through a special training programs for dedicated to urban and rural practitioners conducted by the Government towards appraising unlicensed traditional medicine practitioners (iii) other individuals who qualified for licensing based on other criteria set by the licensing body. It is very essential to bring all practitioners of traditional and alternative medicine into the ambit of the government licensing agency. Proper regulation can only be possible when data on alternative medical practitioners are properly recorded and categorized. Holders of a degree/diploma in CAM from a foreign country who wish to practice alternative medicine in Nigeria should as well, first get themselves registered with the licensing body. Only after getting due permission, they should be allowed to practice. Most of the practitioners of traditional medicine are concentrated in rural areas, effort should be made to reach and educate them so that they can play important roles in primary health care in rural areas. The rural practitioner of alternative medicine should be viewed as an asset rather than a liability to the nation as they can serve people at the grass root level better if properly trained.

## Proposed education program in Traditional Medicine in Nigeria

Proper scientific education and training are important ways to progress forward. The knowledge about traditional medicine has passed from one generation to another orally as there are no written documents^32^. Even if there is some written literature available, there is no proper school to teach or research these subjects. It becomes a real challenge for the practitioners of traditional medicine to preserve the ancestral knowledge that is been passed on from one generation to another verbally.

In this communication, we are proposing a comprehensive six years course curriculum plan for the Doctor of Traditional African Medicine (DTAM). The students who will be graduating in DTAM will learn various courses pertaining to general studies; basic sciences; clinical sciences; CAM practices; traditional and herbal medicine specific to Africa. A six-month compulsory hospital training will be an integral part of the training. For clinical training, the students will be attached to practitioners/experts of traditional medicine. The course curriculum is designed in such a way that the students will have a working knowledge of conventional medicine, CAM, and detailed knowledge of African traditional medicine. The details of the proposed curriculum of the course for DTAM is given in table – 1. Apart from the degree program if a student wishes to learn about TM they should also have the liberty to do 1-year diploma and/or 6 months certificate course on various specialties such as bone setting, midwifery, dentistry, oculist, etc. However, the diploma and certificate (online/offline) should be awarded by some recognized university or organization that is recognized by NUC. Certificates can be also given to the practitioner of TM in the rural areas provided they attend training camps conducted by the Government/NGOs. In special cases, a certificate can be directly given to the person who has many years of experience in that line/area. The National Universities Commission has recently revamped the undergraduate curriculum for Nigerian universities. Complementary and Alternative Medicine studies is included under the Allied Health Sciences as a 4-years B.Sc. degree program.

**Table 1:** Proposed course curriculum for TAM graduation program

Year	Semester I	Semester II
I	English Communication, Use of Library, Study Skills and ICT, Introduction to Computer Elementary Mathematics I, General Biology I, Physics I, Chemistry I, General Biology Practical I Physics Practical I, Chemistry Practical I	Communication in French, Logic Philosophy and Human Existence, Nigerian People and Culture General Biology II, General Chemistry II Elementary Mathematics II, General Physics II General Biology Practical II, Physics Practical II Chemistry Practical II, Introduction to Herbal Medicine
II	Introduction to Entrepreneurship, Leadership Skills, Contemporary Health Issues Complementary and Alternative Medicine Introduction to Pharmacology, Human Physiology & Digestive system, Introduction to Microbiology	Peace Studies and Conflict Resolution, Environment and Sustainable Development, Introduction to Traditional African Medicine, Phytotherapy I, Introduction to Physiotherapy, Introductory Human Anatomy, Medical Microbiology
III	Phytotherapy II, Introduction to Clinical Practice, Nutrition, Diet & Health Promotion, Circulatory System, Basic Gynecology & Obstetrics, Human Nervous system, Human Excretory system	Ethnopharmacology, Pharmacognosy, Toxicology, Principles of Epidemiology & Disease Surveillance, Introduction to Biostatistics, Field Work
IV	Human Genetics, Introduction to Virology, Medical Parasitology, Communicable and Non Communicable Diseases, Research Methodology, Immunology & Immunization, Clinical Training- I (Traditional Medicine)	Clinical Attachment in Hospital
V	Therapeutic Occultism & Spirituality, Surgery and bone-setting, Alternative strategies in Pain management, Gerontology, Medical Investigations & Pathology, Policy Issues in Health Care, Clinical Training II (Traditional Medicine)	Dieting & Therapeutic fasting, Herbs and Cancer Management, Professional Ethics for Community Health, Palliative care in Terminal illnesses, Thermotherapy, Acupuncture, Clinical Training III (Traditional Medicine)
VI	Massage Therapy, Therapeutic Exercise & Yoga, Hydrotherapy, Emergency Medicine, Radiant Healing therapy	Pharmacovigilance of Drugs of Natural Origin, Pharmacoeconomics, Forensic Pharmacy and Pharmacy Management, Research Project

The students after completing graduation studies in TM can go for master's degree program that should comprise of 2 years, 4 semester program with thesis. A student should have the liberty to choose from various established alternative medicine practices as specialization. After successfully completing the course the students will be awarded a master's degree in Traditional Medicine with a specialization in any of the following discipline: Traditional African Medicine; Ayurveda & Yoga; Traditional Chinese Medicine; Homeopathy; Naturopathy; Chiropractic etc. After completion of master's program one can go for Ph.D. program in Tradition Medicine as per the guideline laid by the National Universities Commision. The detailed flowchart is given in figure - 1.

## Conclusion

Health along with education and income plays a key role in determining the Human Development Index (HDI) of a country 33. Presently, Nigeria positioning at 161 out of 189 countries in HDI with a score of 0.539 requires a lot of effort to increase the score. Health inequality is a real challenge for low-income countries^34^. Health inequalities are greatly influenced by income and education status; it has been observed that mortality rates are lower among individuals of higher socio-economic status and just the reverse in lower socio-economic status individuals. Inadequate access to health services is one of the components of rural poverty 35. Modern health care infrastructure seldom exists in deep rural pockets, and even if the facilities are available affordability becomes an issue as result acceptability is poor^36^. So, the next best alternative health care option is traditional medicine 37. So far very little attention is given to properly legitimizing and recognizing the role of traditional medicine in the health care sector of Nigeria. There is an immediate need to start proper education and clinical research on traditional medicine to produce well-trained practitioners of traditional medicine who can practice TM with authority and confidence. In any branch of study, be it arts, science or commerce cannot progress without great teachers, who can work dedicatedly without the greed of materialistic benefits; however, it is also the job of the government to keep these teachers happy by providing them all the assistance and incentives they need so that they can perform to their fullest potential in grim situations^38^. The already existing field practitioners of TM should undergo Government sponsored training to update their knowledge so that they can confidently practice and communicate with other healthcare professionals of modern medicine. Traditional medicine is viewed as a culture-friendly system 39, and in proper conjunction with conventional medicine may play an important role in primary health care in the future, hence, due attention should be given to this sector. India, China, Japan, South Korea, Malaysia, Australia, the USA, and many other nations have streamlined CAM practices in their countries. Ghana has made tremendous progress to integrate their indigenous traditional medicine into their national health care system 40. Taking the cue from these countries Nigeria should also emulate the same about their indigenous traditional medicine. Standardization the practice and education of traditional medicine across Nigeria is the need of the hour^41^.
